# Major contributors to musculoskeletal pain among children receiving hemodialysis

**DOI:** 10.1007/s00467-025-06964-2

**Published:** 2025-10-16

**Authors:** Doaa Mosad Mosa, Mohamed Taman, Abobakr Abdelgalil, Doaa Shokry Alemam, Mohamed M. Zedan, Mai S. Korkor

**Affiliations:** 1 Department of Rheumatology, Rehabilitation, and physical medicine, Faculty of Medicine, Mansoura University Hospital, Mansoura University, 60 Elgomhoria St, Mansoura, 35516 Egypt; 2 Department of Obstetrics and Gynecology, Faculty of Medicine, Mansoura University Hospital, Mansoura University, Mansoura, Egypt; 3 Department of Pediatrics, Faculty of Medicine, Cairo University, Cairo, Egypt; 4 Department of Public Health and Community Medicine, Faculty of Medicine, Mansoura University, Mansoura, Egypt; 5 Department of Pediatrics, Faculty of Medicine, Mansoura University Children’s Hospital, Mansoura University, Mansoura, Egypt

**Keywords:** Factors, Joint/muscle pain, Chronic kidney disease, Children

## Abstract

**Background:**

Children who are undergoing hemodialysis are vulnerable to musculoskeletal (MSK) abnormalities. These conditions can potentially lead to functional impairments and diminished health-related quality of life. Hence, this study was to explore the prevalence of MSK pain in children receiving hemodialysis and the factors associated with this MSK pain.

**Methods:**

This cross-sectional study was conducted on a group of children undergoing hemodialysis at the nephrology unit, Mansoura University Children’s Hospital, from December 2022 to January 2024. Patient demographics, clinical data, musculoskeletal findings, physical function, quality of life assessment parameters, laboratory data, and Dual Energy X-ray Absorptiometry (DEXA) results were collected. All patients were classified into two groups: patients with MSK pain and those without any MSK pain.

**Results:**

The total number of cases with MSK pain was 32 (64%). The most frequent musculoskeletal manifestations were: osteoporosis/osteopenia (64%), myalgia (64%), cramps (60%), arthralgia/arthritis (32%), and regional pain (32%). MSK pain was independently associated with longer dialysis duration, the presence of comorbidity, childhood health assessment questionnaire results, pediatric quality of life inventory, hemoglobin level, serum uric acid, calcium × phosphate product, and DEXA (Z-score).

**Conclusions:**

Involvement of the MSK system is a common morbidity in children with hemodialysis. Calcium × phosphate product (p = 0.026) and vitamin D level (p = 0.003) were the most significant factors associated with MSK pain in multivariate regression analysis.

**Graphical abstract:**

**Figure:**
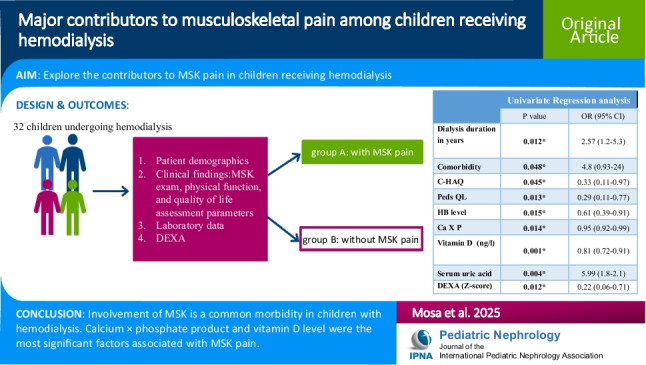
A higher resolution version of the Graphical abstract is available as Supplementary information.

**Supplementary Information:**

The online version contains supplementary material available at 10.1007/s00467-025-06964-2.

## Introduction

Musculoskeletal (MSK) pain is common in children with chronic kidney disease (CKD) undergoing hemodialysis (HD). MSK disorders are the most common cause of MSK pain on long-term hemodialysis. These MSK manifestations may include joints, muscles, bones, linear growth, strength, soft-tissue, and vertebral abnormalities [1, 2].

Various MSK disorders have previously been observed in most patients on HD [3, 4]. These conditions can potentially cause severe disabilities and functional impairments in this fragile group [5]. In addition to osteopenia, osteoporosis, osteosclerosis, amyloidosis, soft-tissue, and vascular calcifications have been noted in long-term HD patients [6, 7].

Furthermore, young children are vulnerable to muscle dysfunction, including muscle atrophy, myopathy, and tendon ruptures, especially at the quadriceps, patellar, and Achilles tendons. The abnormalities of these muscle–tendon units reduce physical functioning in pediatric patients undergoing dialysis [8].

Overall, MSK pain was reported by two-thirds of patients with CKD [7]. It is often not possible to completely alleviate this pain. Still, reduction of this pain to levels where function is not adversely affected is the treatment target in childhood-onset CKD [9].

This study aimed to explore the prevalence of MSK pain and its associated disorders among children on hemodialysis, in addition to studying the factors associated with the development of MSK pain.

## Methods

### Study design

This cross-sectional study was conducted on a group of children undergoing hemodialysis at the nephrology unit, Mansoura University Children’s Hospital from December 2022 to January 2024.

### Patients

Inclusion criteria: All patients with kidney failure on HD and younger than 18 years were included. Exclusion criteria: Patients with acute kidney injury, those with rheumatic disorder prior to dialysis, and those with pain due to surgery, trauma, or skeletal deformity were excluded.

### Work plan

Dialysis was performed three times weekly using 3–3.5 mEq/l dialysate calcium. The duration of each dialysis was 4 h. Data were collected from our patients' files with rheumatologist evaluation as a one-visit assessment and interpreted with respect to the demographic, clinical, disease assessment parameters, laboratory findings, and imaging done according to patients’ conditions as follows:

1. Clinical and demographic features: age, gender, body mass index (BMI), height for age, Tanner stage, CKD duration, interval between CKD and dialysis, dialysis duration, underlying causes, presence of comorbidity, and medications.
2. In addition, the following musculoskeletal findings were assessed by a rheumatologist with a thorough MSK examination as a single-visit assessment: fibromyalgia syndrome, myalgias, arthralgia/arthritis, tenosynovitis, cramps (painful involuntary contractions), ectopic calcifications, carpal tunnel syndrome, spondyloarthritis, fracture, rotator cuff syndrome, crystal arthropathy/chondrocalcinosis, and dialysis-related amyloidosis.
3. Pain assessment: site, pain severity rating using a 10-point numerical rating scale (NRS), from 0 (no pain) to 10 (very painful); mild/moderate/severe, 0–3/10, 4–6/10, 7–10/10, respectively [9].
4. Physical function assessment was done by the Childhood-Health Assessment Questionnaire (C-HAQ). There were eight groups of questions on activities of daily living included (dressing and grooming, arising, feeding, walking, hygiene, reach, grip, and common daily activities), and the total score was 0–3 [10]. The quality of life assessment was performed by the Pediatric Quality of Life Inventory (Peds-QL), which measures the health-related quality of life in children, covering four Core Scales (Physical, Emotional, Social, School Functioning) [11].
5. The following laboratory parameters were assessed: blood urea nitrogen (BUN) (mg/dL), serum creatinine (mg/dL), estimated glomerular filtration rate (eGFR) (mL/min/1.73 m^2^). Additionally, we measured hemoglobin (g/dL), albumin (g/dL), calcium (Ca) (mg/dL), phosphate (P) (mg/dL), calcium × phosphate (Ca × P) product (mg/dL), vitamin D level (ng/L), parathyroid hormone (PTH) (pg/mL), and serum uric acid levels (mg/dL).
6. Osteoporosis/osteopenia assessment by Dual Energy X-ray Absorptiometry (DEXA). Other imaging modalities, such as plain X-ray or ultrasound, were used according to the patients’ presentations.

All patients were classified into two groups based on the presence of MSK pain: group A, with any MSK pain, and group B, without any MSK pain.

### Statistical analysis

Data were analyzed using the Statistical Package of Social Science (SPSS) program for Windows (Standard version 24). The normality of data was first tested with a one-sample Kolmogorov–Smirnov test. Qualitative data were described using numbers and percentages. The association between categorical variables was tested using the Chi-square test, while Fisher's exact and Monte Carlo tests were used when the expected cell count was less than 5. Continuous variables were presented as mean ± SD (standard deviation) for normally distributed data and median (Min–Max) for non-normal data.

The two groups were compared by an independent t-test (parametric) and a Mann–Whitney test (non-parametric). Spearman correlation was used to correlate continuous data. Significant variables on univariate analysis were entered into the logistic regression model using the forward Wald statistical technique to detect the most significant determinants and to control for possible interactions and confounding effects. The results were considered significant when p ≤ 0.05.

### Ethical considerations

Informed consent was obtained from each patient/parent before the procedures. The study was conducted after the approval of the Institutional Research Board (IRB) of the Faculty of Medicine, Mansoura University, Egypt.

## Results

### Characteristics of the study group

We enrolled 50 patients; their demographic data, disease characteristics, and the underlying etiologies of CKD are displayed in Table 1. The majority of cases were males (58%), and their mean age was 11.46 ± 3.93SD. The median of CKD duration, dialysis duration, and the interval between CKD and dialysis onset was 3, 1.3, and 1 years, respectively. Most cases had growth failure (low height-for-age) (80%) and a delayed puberty onset of pubic hair (the most common Tanner stages were stages 1 and 2). Congenital anomalies of the kidney and urinary tract (CAKUT) (60%) and acquired conditions (24%), such as glomerulonephritis or tubulointerstitial nephritis, were the two most common causes of CKD among the children, as exhibited in Table 1.

**Table 1:** Characteristics of the studied group

	The studied group (n = 50)
Age (years)	
Mean ± SD	11.46 ± 3.93
Sex	
Male Female	29 (58%) 21 (42%)
BMI	
Mean ± SD	17.16 ± 5.39
Underlying disease	
CAKUT Acquired conditions • Glomerulonephritis • Tubulointerstitial nephritis • Diabetic nephropathy Genetic conditions • Atypical HUS • Alport syndrome • Cystinosis • Hyperoxaluria Unknown etiology	30 (60%) 12 (24%) 9 (18%) 2 (4%) 1 (2%) 7 (14%) 3 (6%) 1 (2%) 1 (2%) 2 (4%) 1 (2%)
CKD duration in years	
Median (Min–Max)	3 (0.5–7)
Dialysis duration in years	
Median (Min–Max)	1.3 (0.2–6)
Interval between CKD and dialysis	
Median (Min–Max)	1 (0–5.9)
Children with growth failure (low height-for-age)	40 (80%)
Tanner stage of pubic hair	
1 2 3 4	25 (50%) 17 (34%) 4 (8%) 4 (8%)

*CKD *chronic kidney disease, *BMI *body mass index; HUS, hemolytic uremic syndrome, *CAKUT *congenital anomalies of the kidney and urinary tract

Unlike in adults where glomerular disease, hypertension, and diabetes are predominant etiologies, CAKUT includes aplastic, hypoplastic, and dysplastic kidneys, obstructive uropathy as posterior urethral valves (PUV) and Pelvi-ureteric junction obstruction (PUJO), vesicoureteral reflux (VUR), polycystic and multicystic dysplastic kidneys (MCDK), hydronephrosis, duplex kidney, duplicated collecting system and megaureter

### The musculoskeletal findings and assessment parameters among the studied cohort

The total number of cases with MSK manifestations was 32 (64%). The most frequent musculoskeletal manifestations were: osteoporosis/osteopenia (64%), myalgia (64%), cramps (60%), arthralgia/arthritis (32%), and regional pain (32%). On the other hand, the least frequent presentations were: fracture (2%) and avascular necrosis (2%). No cases of dialysis-related spondyloarthropathy or amyloidosis were observed in our cohort (Table 2).

**Table 2:** Musculoskeletal manifestations, assessment parameters, and treatment among patients on hemodialysis

	The studied group (n = 50)
No. of cases with musculoskeletal manifestations	32 (64%)
Osteoporosis/osteopenia	32 (64%)
Myalgia	32 (64%)
Cramps	30 (60%)
Arthralgia/arthritis	16 (32%)
Regional pain (pain localized to a specific area of the body, such as shoulder or pelvic girdle)	16 (32%)
Fibromyalgia syndrome	10 (20%)
Tenosynovitis	3 (6%)
Ectopic calcifications	2 (4%)
Carpal tunnel syndrome	2 (4%)
Rotator cuff syndrome	2 (4%)
Crystal arthropathy/chondrocalcinosis	2 (4%)
Fracture	1 (2%)
Avascular necrosis	1 (2%)
Pain Site	
Generalized Upper limb Lower limb Back and lower limb	25 (50%) 1 (2%) 3 (6%) 1 (2%)
Pain severity rating Mild Moderate Severe	29 (58%) 15 (30%) 6 (12%)
C-HAQ Median (Min–Max)	0.4 (0.1–2.8)
Peds-QL Median (Min–Max)	1.9 (0.1–3.5)
Comorbidity	14 (28%)
Treatment response#	
No response Good response	23 (46%) 27 (54%)

*C-HAQ *Childhood Health Assessment Questionnaire, *Peds-QL *Pediatric Quality of Life Inventory

#Main treatment lines used for MSK pain include: analgesics, muscle relaxant, and calcium supplements

Comorbidity: cardiomyopathy or liver impairment

The most common site of MSK pain was generalized bone and joint pain. Most of our patients experienced mild pain on the severity rating. The median of C-HAQ was 4, whereas the median of quality of life as assessed by Peds-QL was 1.9 (Table 2).

### Treatment among patients on hemodialysis

Most of our cases received treatment lines for pain with simple analgesics, muscle relaxants, and calcium supplements. However, patients who had already been diagnosed with gout, for example, received anti-hyperuricemics and a short course of nonsteroidal anti-inflammatory drugs (NSAIDs). Cases with significant tenosynovitis or joint pain received a short course of NSAIDs as well. Physiotherapy sessions were prescribed to some patients based on clinical judgment.

### The demographic and clinical differences between the studied groups

The comparison of the main patient characteristics between the groups is shown in Table 3. Group A is more likely to have longer dialysis duration, associated comorbidities, higher C-HAQ, and Ped-QL. However, no significant difference was observed between the two groups regarding other parameters.

**Table 3:** The main characteristic differences between the two studied groups

	Group A Musculoskeletal pain (n = 32)	Group B No musculoskeletal pain (n = 18)	Test of significance	P value
Age (years)				
Mean ± SD	11.75 ± 3.92	10.94 ± 4.03	t = 0.690	0.49
Sex				
Male Female	16 (50%) 16 (50%)	13 (72.2%) 5 (27.8%)	χ^2^ = 2.34	0.12
CKD duration in years	3 (0.5- 7)	4 (0.5—7)	Z = 0.540	0.58
Dialysis duration in years	2 (0.2- 6)	0.9 (0.2- 3)	Z = 2.62	0.009*
Interval between CKD and dialysis	1.2 (0- 5.9)	1 (0- 5)	Z = 0.193	0.84
BMI	16.8 ± 3.8	17.7 ± 7.5	t = 0.535	0.59
Children with stunted growth	29 (90.6%)	11 (61.1%)	χ^2^ = 2.78	0.59
Tanner stage of pubic hair				
1 2 3 4	16 (50%) 13 (40.6%) 0 (0%) 4 (12.4%)	9 (50%) 4 (22.2%) 4 (22.2%) 0 (0%)	MC	0.15
Comorbidity	3 (9.4%)	2 (11.1%)	χ^2^ = 3.97	0.046*
C-HAQ	0.45 (0.1- 2.8)	0.4 (0.1- 1.8)	Z = 2.09	0.037*
Peds QL	1.9 (0.3- 3.5)	1.8 (0.1- 3)	Z = 2.86	0.004*

*CKD *chronic kidney disease, *BMI *body mass index, *C-HAQ *Childhood Health Assessment Questionnaire, *Peds-QL *Pediatric Quality of Life Inventory

Comorbidity: cardiomyopathy or liver impairment. *p < 0.05 is statistically significant

### Laboratory parameters and DEXA score difference between the two studied groups

There was a statistically significant difference between children with MSK pain versus those without regarding hemoglobin level, calcium x phosphate product, vitamin D, serum uric acid, and Z-score of DEXA scan, as displayed in Table 4.

**Table 4:** Laboratory parameters and DEXA (Z-score) differences between the two studied groups

	Group A Musculoskeletal pain (n = 32)	Group B No Musculoskeletal pain (n = 18)	Test of significance	P value
HB level (g/dL)	10.3 ± 1.73	11.6 ± 1.41	t = 2.73	0.009*
BUN (mg/dL)	17 (10- 60)	20.7 (7- 60)	Z = 0.314	0.75
Serum creatinine (mg/dL)	9.87 ± 2.76	9.51 ± 2.83	t = 0.439	0.66
eGFR (mL/min/1.73 m^2^)	5.69 ± 2.00	5.66 ± 1.56	t = 0.062	0.95
Serum albumin (g/dL)	**3.9 ± 0.27**	3.84 ± 0.38	t = 0.688	0.49
Serum total calcium (mg/dL)	8.87 ± 0.98	9.05 ± 0.93	t = 0.644	0.52
Serum phosphate (mg/dL)	5.81 ± 2.26	5.59 ± 1.91	t = 0.354	0.72
Ca X P product (mg/dL)	60.4 ± 18	45.9 ± 17.2	t = 2.79	0.008*
PTH (pg/mL)	653 (212- 2827)	560 (87- 3664)	Z = 1.11	0.26
Vitamin D (ng/l)	23.5 ± 8.24	34.89 ± 8.88	t = 4.56	≤ 0.001*
Lipid profile				
Normal Elevated	18 (56.2%) 14 (43.8%)	12 (66.7%) 6 (33.3%)	χ^2^ = 0.521	0.47
Serum uric acid (mg/dL)	5.70 ± 0.85	4.92 ± 0.57	t = 3.46	0.001*
ESR (mm/hr)	6 (5- 30)	5 (5- 35)	Z = 0.790	0.42
DEXA (Z-score)	−2 (−5 −0.5)	−1.5 (−2.2- 1.8)	Z = 3.04	0.002*

*HB *hemoglobin, *BUN *blood urea nitrogen, *eGFR *estimated glomerular filtration rate, *Ca × P product* calcium × phosphate product, *PTH *parathyroid hormone, *ESR *erythrocyte sedimentation rate, *DEXA *dual energy X-ray absorptiometry

*p < 0.05 is statistically significant

### Factors associated with MSK pain by regression analysis

In our study, univariate analysis indicated that MSK pain was significantly associated with longer dialysis duration, the presence of comorbidity, C-HAQ, Peds QL, hemoglobin level, calcium x phosphate product, serum uric acid, and DEXA (Z-score). However, after multivariate analysis, MSK pain was only associated with calcium × phosphate product and vitamin D level (Table 5).

**Table 5:** Factors associated with MSK pain by regression analysis

	Univariate Regression analysis		Multivariate Regression analysis	
	P value	OR (95% CI)	P value	OR (95% CI)
Dialysis duration in years	0.012*	2.57 (1.2–5.3)	-	-
Comorbidity	0.048*	4.8 (0.93–24)	-	-
C-HAQ	0.045*	0.33 (0.11–0.97)	-	-
Peds QL	0.013*	0.29 (0.11–0.77)	-	-
HB level (g/dL)	0.015*	0.61 (0.39–0.91)	-	-
Ca X P (mg/dL)	0.014*	0.95 (0.92–0.99)	0.026*	0.95 (0.91–0.99)
Vitamin D (ng/L)	0.001*	0.81 (0.72–0.91)	0.003*	0.83 (0.74–0.94)
Serum uric acid (mg/dL)	0.004*	5.99 (1.8–2.1)	**-**	
DEXA (Z-score)	0.012*	0.22 (0.06–0.71)	-	

*CI *confidence interval, *OR *odds ratio, *C-HAQ *Childhood Health Assessment Questionnaire, *Peds-QL *Pediatric Quality of Life Inventory, *HB *hemoglobin, *Ca × P product* calcium × phosphate product, *DEXA *dual energy X-ray absorptiometry

Comorbidity: cardiomyopathy and liver impairment. *p < 0.05 is statistically significant

## Discussion

Our results showed that among 50 enrolled children, more than half of our patients (64%) had musculoskeletal presentations, similar to El-Najjar et al. [7] (60.4%) and Hsu et al. [12] (53.3%) in their adult studies.

The most common MSK presentations were osteoporosis/osteopenia, myalgia, cramps, arthralgia/arthritis, and regional pain. These findings were similar to previous reports [7, 12–15]. However, Afifi et al. [4] described a high prevalence of arthralgia among their cohort (83%), which could be attributable to the longer HD duration in their study.

Focusing on the prevalence of osteopenia/osteoporosis among our children, it was recorded in 32% cases. A similar study reported osteopenia in 37.2% [16]. This was not in agreement with Vachtenheim et al. [17] and Polymeris et al. [18] who reported a lower prevalence of osteoporosis, 12.9% and 14.3%, respectively. In contrast, Afifi et al. [4] detected osteopenia in 56.6% of patients, followed by osteoporosis (24.5%). Further, Joker et al. [13] noted osteopenia in 43.2% of their cases. The variation in osteopenia/osteoporosis prevalence among these studies may be due to the difference in population demographics, other patient-related risk factors, or dialysis and follow-up duration.

The other MSK disorders that were found in our cohort include: fibromyalgia syndrome (20%), carpal tunnel syndrome (4%), and fracture (1%). Fibromyalgia was reported in a higher prevalence (51%) in a previous study [14] that included adult cases with a high rate of associated comorbidities. Likewise, carpal tunnel was recorded at a higher rate (10.4%−31.7%) in preceding reports [7, 14, 19, 20] that was found to be associated with longer duration of dialysis as stated by Kopeć et al. [21].

Concerning the prevalence of tenosynovitis (6%) and rotator cuff syndrome (4%) in our group, it was close to the study of El-Najjar et al., who stated that tendonitis occurred in 6.9% of their patients [7]; Rillo et al. documented tendon injury in 3.6% of cases [22]. The pathophysiology of these manifestations is not clearly elucidated, but crystal, electrolytes overloads, or peri-tendinous depositions may play a role [7].

Ectopic calcifications and crystal-associated arthropathy were reported at the same rate (4%) in our cases, which in agreement with [7, 17] findings. It seems that the incidence of gout after initiation of dialysis is rare [23]. No cases with dialysis-related amyloidosis or spondyloarthropathy were reported in our cohort. However, these findings were described in 8% of HD patients by Gheita et al. [14], a study that was conducted on adult patients with long dialysis duration.

As expected, children who reported MSK pain tend to have longer dialysis duration and comorbidities, as reported in our cohort, and in the cohorts of Hsu et al. [12] and Lim et al. [24]. Furthermore, in our study, the health assessment questionnaire (C-HAQ) and quality of life (Peds-QL) indices exhibited a significant difference between both groups, with higher values in cases with MSK pain, in concordance with preceding studies [4, 7]. Children with CKD who are undergoing HD have progressive deficits of their functional muscle–bone unit and chronic pain that induce physical function and health-related quality of life impairment [9].

Regarding the laboratory differences among the studied groups, children with chronic MSK pain showed higher calcium × phosphate product and serum uric acid, but lower hemoglobin and vitamin D levels. Elevated serum uric acid is an expected consequence of kidney function impairment, oxidative stress, or diuretic use [25, 26].

Moreover, imbalance of electrolytes, such as calcium, phosphate, and vitamin D deficiency with hyperparathyroidism, are considered key contributors to kidney bone disease [27]. During kidney disease, the progressive deficiency of active vitamin D and the decrease in the number of vitamin D receptors directly affect the MSK system function [28]. Hence, hyperuricemia, increased calcium × phosphate product, and low vitamin D were significantly associated with MSK pain, as stated in previous studies as well [4, 7, 13, 29].

Despite a higher level of PTH in cases with MSK pain, there was no statistically significant difference between the 2 groups. El-Najjar et al. [7] and Golan et al. [30] found that a higher PTH level was associated with chronic pain in hemodialysis patients. Phosphate retention can provoke secondary hyperparathyroidism in CKD cases [28].

In our study, MSK pain was significantly associated with longer dialysis duration, the presence of comorbidity, C-HAQ, Peds QL, hemoglobin, vitamin D levels, calcium × phosphate product, serum uric acid, and DEXA (Z-score) as revealed from the univariate analysis; these findings correspond with those of Hsu et al. [12]. All these factors seem to be the major contributors to MSK pain as stated previously [29, 31]. Additionally, in the current study, multivariate analysis showed that the calcium × phosphate product and vitamin D level were the most significant contributors to MSK pain, which are crucial factors to be considered while managing patients with chronic MSK pain.

Our study had some limitations; The small sample size hinders the correlation tests, MSK complaints are sometimes under expressed by children, and the full imaging information, such as plain X-ray or MSK ultrasound, was lacking. Further, there were some potential unmeasured contributors such as inflammatory mediators, psychological factors, or uremic toxins, that need in-depth analysis in long-term studies. However, this study provides a useful start for future studies that would explore the main contributing parameters of chronic MSK manifestations for better understanding and management of chronic MSK pain in young children undergoing kidney dialysis.

## Conclusion

Involvement of the MSK system is a common morbidity in children with hemodialysis that diminishes their physical function and quality of life. This present study demonstrated that MSK pain was independently associated with longer dialysis duration, the presence of comorbidity, C-HAQ, Peds QL, hemoglobin level, serum uric acid, DEXA (Z-score), and especially vitamin D level, and calcium × phosphate product.

## Supplementary Information

Below is the link to the electronic supplementary material.

Graphical abstract (PPTX 88.2 KB)

## Data Availability

All data generated during this study are in this article.
