# Supplementary material for: Major contributors to musculoskeletal pain among children receiving hemodialysis
Source: Pediatr Nephrol. 2025 Oct 16;41(2):539–46. doi: 10.1007/s00467-025-06964-2 (PMC12727712; doi:10.1007/s00467-025-06964-2)
Supplement: Supplementary file 1 — Graphical abstract (PPTX 88.2 KB) [file 467_2025_6964_MOESM1_ESM.pptx]

## Slide 1
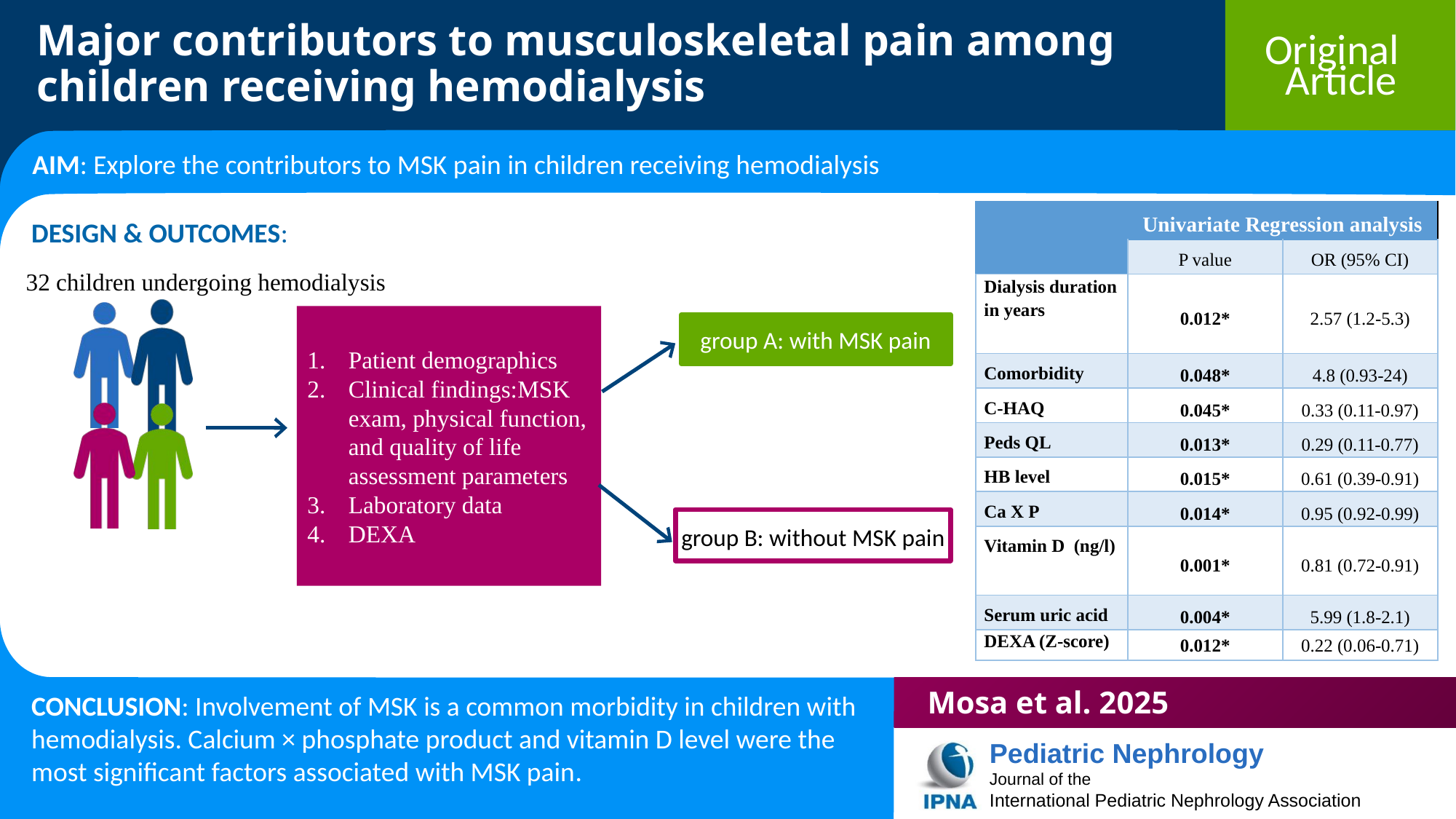

Major contributors to musculoskeletal pain among children receiving hemodialysis
AIM: Explore the contributors to MSK pain in children receiving hemodialysis
| | Univariate Regression analysis | |
| --- | --- | --- |
| | P value | OR (95% CI) |
| Dialysis duration in years | 0.012\* | 2.57 (1.2-5.3) |
| Comorbidity | 0.048\* | 4.8 (0.93-24) |
| C-HAQ | 0.045\* | 0.33 (0.11-0.97) |
| Peds QL | 0.013\* | 0.29 (0.11-0.77) |
| HB level | 0.015\* | 0.61 (0.39-0.91) |
| Ca X P | 0.014\* | 0.95 (0.92-0.99) |
| Vitamin D (ng/l) | 0.001\* | 0.81 (0.72-0.91) |
| Serum uric acid | 0.004\* | 5.99 (1.8-2.1) |
| DEXA (Z-score) | 0.012\* | 0.22 (0.06-0.71) |
DESIGN & OUTCOMES:
32 children undergoing hemodialysis
Patient demographics
Clinical findings:MSK exam, physical function, and quality of life assessment parameters
Laboratory data
DEXA
group A: with MSK pain
group B: without MSK pain
Mosa et al. 2025
CONCLUSION: Involvement of MSK is a common morbidity in children with hemodialysis. Calcium × phosphate product and vitamin D level were the most significant factors associated with MSK pain.
